# Effect of Forward Reaching With a Modified Sitting Position on Muscle Contraction in the Paretic Lower Extremity of Individuals in the Early Sub-Acute Phase of Stroke: A Randomized Control Trial

**DOI:** 10.7759/cureus.66998

**Published:** 2024-08-16

**Authors:** Tluway B Nuwagi, Sivakumar Ramachandran, C.M Radhika

**Affiliations:** 1 Department of Physiotherapy, Sri Ramachandra Institute of Higher Education and Research, Chennai, IND

**Keywords:** quadriceps muscle, tibialis anterior, stroke, rehabilitation, postural control, forward reach

## Abstract

Introduction

Forward reaching in sitting has been shown to facilitate muscle contraction in the paretic lower limb of stroke survivors. Change in the sitting surface has been shown to alter the contribution of lower extremity muscles to maintain postural control. This study investigated the effect of forward reaching in a modified sitting position on the paretic lower extremity muscles of patients with stroke.

Methods

First-time cerebral stroke survivors in their early sub-acute phase were randomly allocated to the experimental or control group. The experimental group engaged in training sessions focusing on reaching a target while seated with only the paretic foot placed on a support, whereas the control group performed the same task with both feet supported on the surface. Each group completed three sets of 10 repetitions of forward reaching for eight days as a part of the training. Quadriceps and tibialis anterior muscle activity in the paretic leg were measured using surface electromyography before the first and after the last session of intervention. Statistical analysis was conducted using parametric tests with a significance level set at p < 0.05.

Results

Sixty-three subjects completed the study, with 31 in the experimental group and 32 in the control group. The results of the post-intervention analysis indicated a statistically significant increase in the EMG activity of the tibialis anterior and quadriceps muscle surfaces in both groups (p < 0.001). Notably, the experimental group exhibited significantly higher muscle activity in both quadriceps and tibialis anterior compared to the control group (p < 0.001).

Conclusion

Forward reaching with only the paretic lower limb grounded effectively improves quadriceps and tibialis anterior muscle recruitment in the early sub-acute phase of stroke.

## Introduction

Stroke is the second leading cause of death and the third leading cause of combined death and disability worldwide. According to the World Stroke Organization (WSO), there has been a global increase in the burden, cases, and deaths from stroke between 1990 and 2019 [1]. Various therapeutic methods have been tested to enhance motor control in the lower extremities, including robotics, virtual reality, functional training, and circuit class training [2,3].

Functional training is believed to promote neuroplastic changes. Generally, functional training involves activities wherein the individual has to use a group of muscles to achieve a goal. The participants will be made to focus on the goal and quality of movement rather than individual muscle contraction. For instance, a commonly used activity is reaching in sitting, which necessitates the engagement of lower extremity muscles to stabilize the support base during reaching [4,5]. Research indicates that forward-reaching tasks can activate multiple muscle groups in the lower extremities, presenting potential therapeutic benefits for individuals with stroke [6,7]. In particular, the anterior tibial muscle in the paretic lower limb is activated when sitting, and reaching is performed with the foot grounded on a support surface [8-10].

Sivakumar et al. (2019) found that altering the sitting surface while reaching can influence the contraction of the anterior tibial muscle in the paretic lower extremity [11]. Studies on the biomechanics of reaching forward in stroke survivors have underscored that adjustments in sitting posture, such as altering trunk angles or foot positions, can impact muscle activation sequences [12,13].

We assumed that the more effective muscle contractions in the paretic lower extremity could occur when a post-stroke individual grounds the paretic lower extremity exclusively during reaching the task. It was hypothesized that since the normal extremity is not grounded, during the task, the paretic lower extremity is likely to be forced to stabilize the base, thereby enhancing its muscle contraction. Consequently, this study aimed to investigate the impact of forward-reaching tasks in a modified seated position wherein the affected lower limb is solely grounded, on selected muscles in the paretic lower extremity.

## Materials and methods

Study period and duration

The study was conducted from March 2024 to May 2024.

Study design

This is a randomized control trial with assessor blinding. The study was approved by the institutional ethics committee with registration number CSP/23/DEC/140/923 and registered in the clinical trial registry of India (CTRI/2024/03/063569).

Inclusion criteria

Individuals who had experienced the first occurrence of cerebral hemispheric middle cerebral artery (MCA) territory stroke with infraction, displaying a motor recovery of Brunnstrom stage 2 or below in the lower extremity, and capable of independent sitting on the bed were identified for potential inclusion.

Exclusion criteria

Those with hemorrhagic stroke and cognitive impairments impeding their ability to comprehend instructions and participate in the study were not included in the study.

Data collection

Participants were recruited from the neuro-medicine ward of a tertiary care university teaching hospital. Informed consent was obtained from participants after explaining the procedure. 

Sample size calculation

The sample size was determined based on data from a pilot study involving 10 stroke survivors who performed forward reaching in a sitting position for eight sessions of therapy. Surface electromyography (SEMG) was recorded from the tibialis anterior muscle pre- and post-training. The difference in the average SEMG value for the tibialis anterior was utilized for sample size calculation with a priori for hypothesis testing. An alpha of 0.05 and a power of 0.95 were used for calculation. A sample size of 30 participants per group arrived, and accounting for 10% attrition, 35 participants per group were finalized.

Randomization and training

**Figure 1:**
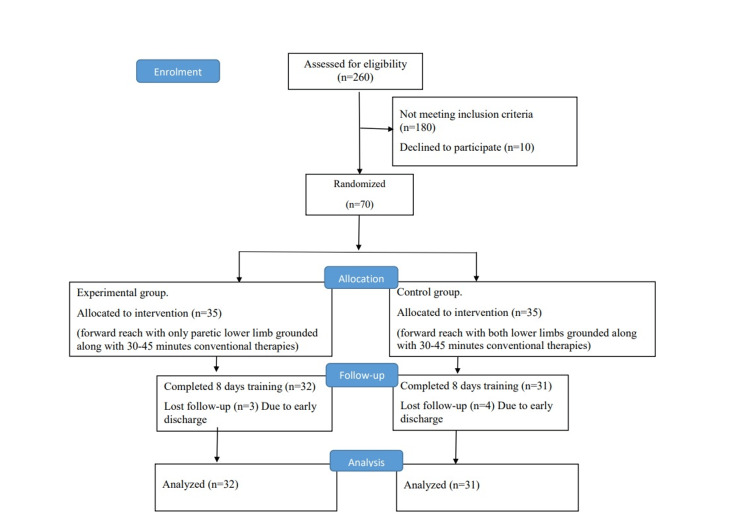
Consort flow for randomization and intervention

**Figure 2:**
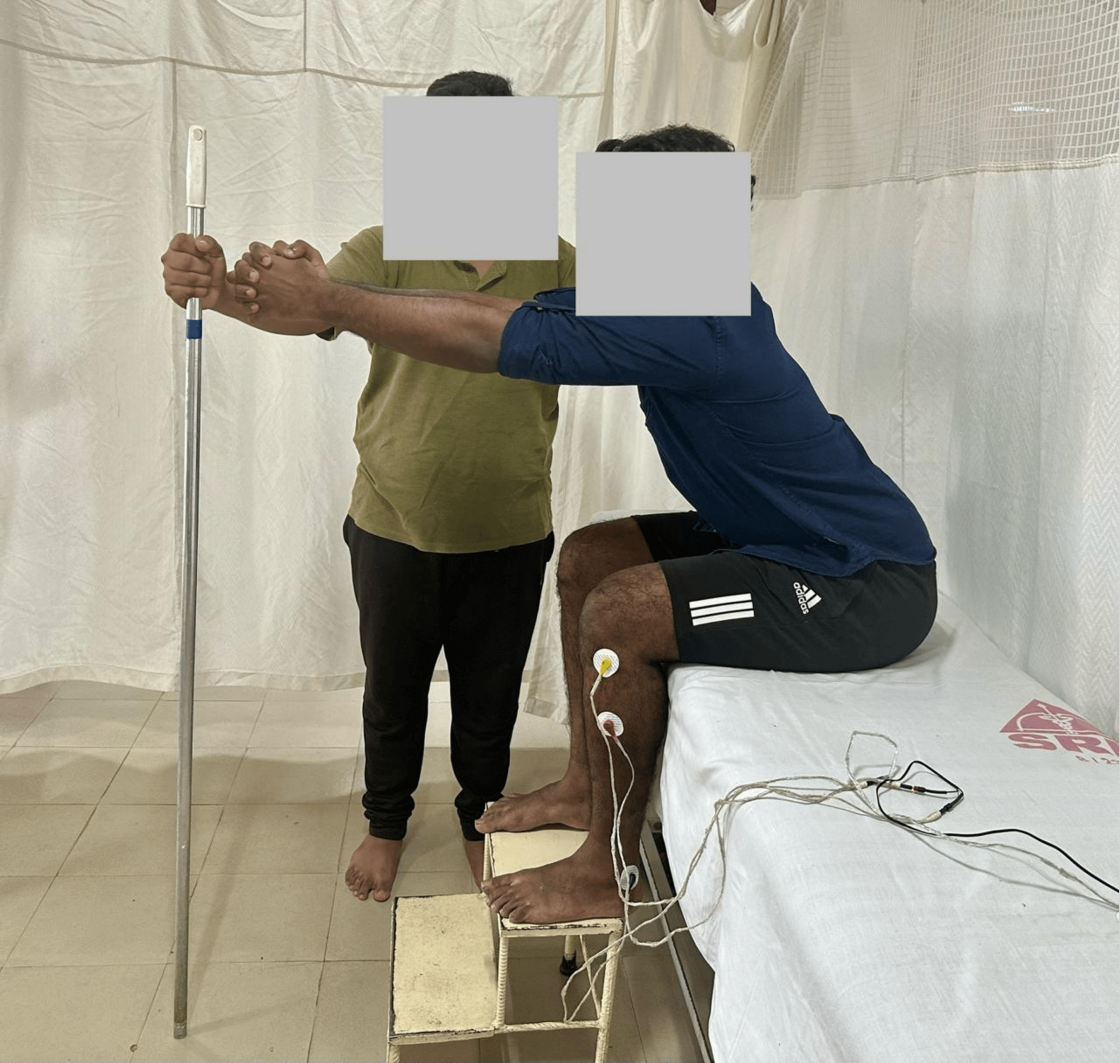
Participant in the control group reaching the target while sitting on the bed with his feet supported on the footstool

**Figure 3:**
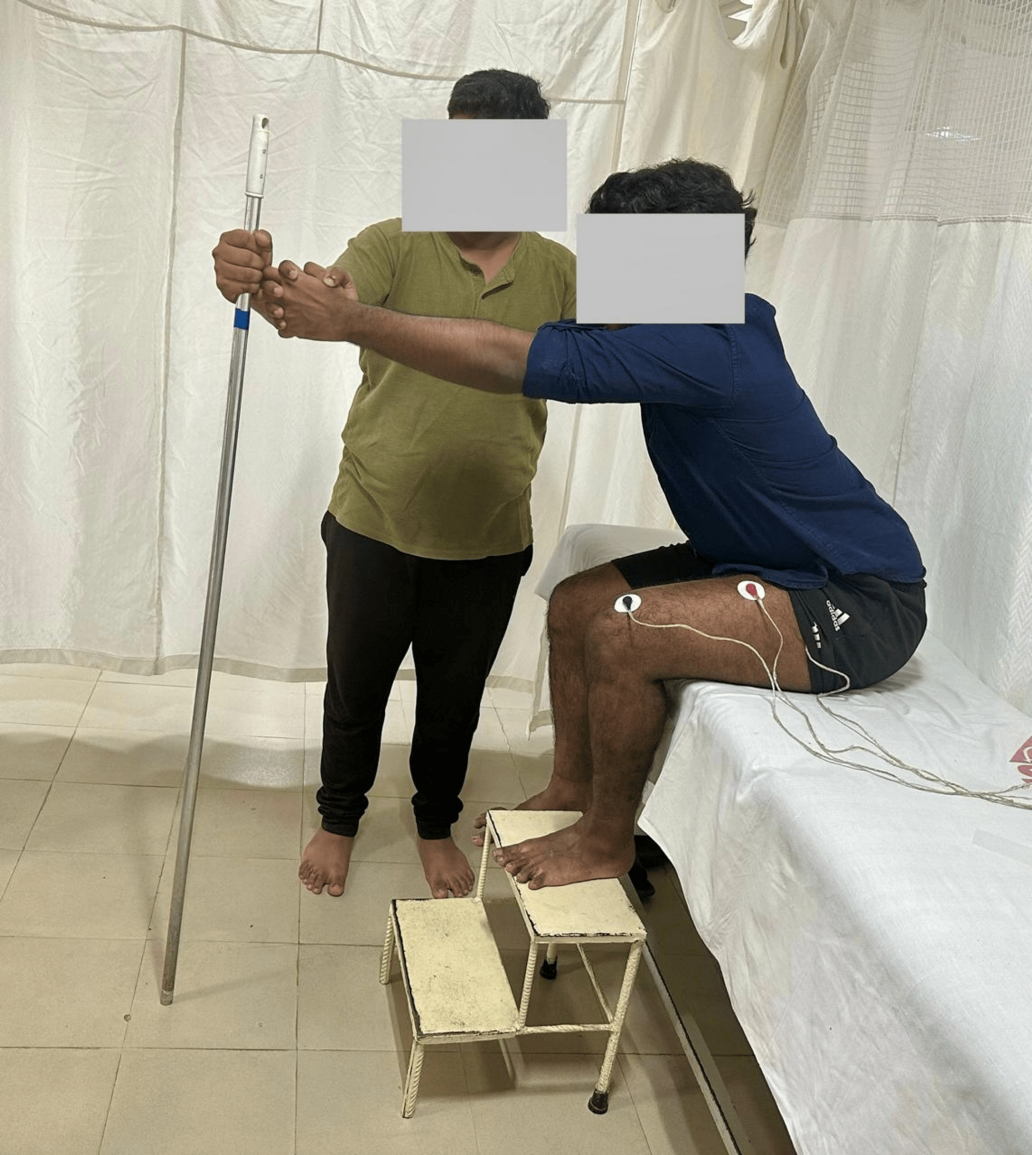
Participant in the experimental group reaching while sitting with only the affected foot placed on the footstool

Participants were allocated randomly to the control and experimental groups using a block randomization size of 4 (Figure 1). Both groups underwent 30 to 45 minutes of physiotherapy, encompassing exercises for the upper limb, trunk, and lower limb. The control group performed reaching training with both feet supported on a stool while sitting on the bed (Figure 2). The experimental group performed reaching training with only the affected foot placed on the stool while sitting on the bed and the unaffected foot left unsupported (Figure 3). All the participants were instructed to perform reaching in the sagittal plane at shoulder level. The reach distance was standardized as a length equal to one-and-half of the arm length measured from the acromion process to the tip of a middle finger of the unaffected upper limb. The unaffected and affected upper extremities were clasped together while reaching the target. Assistance on the affected upper limb was given to support the upper limb whenever required during reaching. Ten times reaching was repeated for one set and three such sets were done for each session of physiotherapy. All the participants underwent eight sessions of therapy over eight days. 

Outcome measure 

Baseline and post-training SEMG was recorded from the tibialis anterior and quadriceps muscle on the affected side. Neurotrac software 4.0 from VM (Verity Medical Ltd, United Kingdom) was used for SEMG recording. The placement of electrodes followed established guidelines (www.seniam.org). The tibialis anterior was selected as the muscle of interest due to previous research highlighting its contribution to sitting and reaching tasks. SEMG from the tibialis anterior was recorded with the active electrode over the muscle bulk, the reference electrode positioned 4 cm proximally, and the ground electrode over the lateral malleolus. The quadriceps muscle was chosen due to its role in stabilizing the knee during forward weight shifting. SEMG was recorded from the rectus femoris muscle of quadriceps. The activity was recorded with electrodes placed halfway along the line from the anterior superior iliac spine to the superior part of the patella whereby the active electrode was placed at the muscle bulk, a reference electrode was placed 2 cm proximally, and the ground electrode at the supra-patella tendon. The recording was done when the participant reached forward toward the target at a one-and-half-arm distance. Three recordings were captured during three reaching trials and only the highest of all three average values was considered for analysis.

Data analysis

The average SEMG [µV] was utilized for analysis, with the highest value obtained from the three trials during pre- and post-intervention testing. A paired t-test and independent Student's t-test were used to assess differences within and between the groups. The difference of values in pre-post intervention within each group was considered as the impact of training and the values were utilized for between the group evaluation. The statistical significance was set at p<0.05. The effect size was calculated using Cohen D.

## Results

Sixty-three subjects completed the training when 70 subjects were enrolled in the study (90.0% retention). The experimental and control groups had 32 and 31 participants, respectively. The recruitment was stopped as the minimum sample size of 30 was reached in both groups. After completing the eight-session training program, all 63 participants were included in the final analysis (Table 1).

**Table 1:** Demographic details of the participants and stroke-related parameters

Variables		Experimental group n=32	Control group n=31
Age(mean±SD)		51.67(11.83)	57.71(12.93)
Gender	Male	25(78.13%)	24(77.42% )
	Female	7(21.87%)	7(22.58%)
Paretic side	Right	21(65.6%)	15(48.4%)
	Left	11(34.4%)	16(51.4%)

Participants in both groups showed increased quadriceps and tibialis anterior muscle contraction following training (Table 2). The 95% CI for change in quadriceps muscle SEMG in the experimental group is 8.41 μV to 9.95 μV, and tibialis anterior is 8.56 μV to 10.63 μV. In the control group, the 95% CI for change in quadriceps muscle SEMG is 3.11 μV to 4.02 μV, and the tibialis anterior is 3.93 μV to 5.54 μV. In both groups, it was observed that tibialis anterior activity was more impacted with the training than quadriceps. However, the change in the experimental group was higher than that in the control group in both muscle groups. 

**Table 2:** Average EMG output (mean & SD) from quadriceps and tibialis anterior muscles a: Paired t-test; significant at p≤0.05 b: Independent t-test: significant at p≤0.05 EMG: Electromyography

Mean μV (SD) for the muscles	Experimental n=32				Control group n=31				Differences between pre- and post-intervention			
									experimental	Control	t-value	p-value
	Pre-intervention	Post-intervention	t-value	p-value	Pre-intervention	Post-intervention	t-value	p-value	Pre/post difference	Pre/post difference		
Quadriceps	5.24 (2.81)	14.42 (4.38)	23.28	0.001^a^	4.79 (2.64)	8.37 (2.64)	15.25	0.001^a^	9.18 (2.23)	3.57 (1.30)	12.14	P<0.001^b^
Tibialis anterior	4.42 (2.79)	13.35 (4.80)	18.14	0.001^a^	4.40 (2.10)	9.14 (2.36)	12.97	0.001^a^	9.60 (2.99)	4.74 (2.28)	7.24	P<0.001^b^

We used the difference within the group value rather than post-training raw data to test the difference between the groups. The difference between the groups reached statistical significance. The groups' difference in average micro-volt recorded for quadriceps activity showed 5.61µV (95% CI - 4.69 to 6.53) and for tibialis anterior showed 5.13µV (95%CI - 3.79 -6.47).

The effect size was calculated using Cohen's d. The effect size in the control and experimental group was larger. In the experimental group Cohen's d for quadriceps was 4.11 and for tibialis anterior was 3.21, while in the control group, the effect size for quadriceps was 2.75 and for tibialis anterior was 2.79. The effect size reveals a better treatment effect in the experimental group compared to the control group. 

## Discussion

This study investigated the effect of forward reaching in a modified sitting position on contractions of selected muscles in the paretic lower extremities of stroke survivors. We found that grounding the paretic lower extremity only on the support while reaching forward increased the contribution of the paretic lower extremity for the control of forward momentum. In the present study, we tested the tibialis anterior and quadriceps muscles for their activity following training by reaching forward with both lower extremities on the ground and only the paretic lower extremity on the ground. The training where only the paretic lower extremity alone was grounded led to a greater activity of the tibialis anterior and quadriceps muscle than training with both lower extremities grounded while reaching. 

The tibialis anterior muscle plays a crucial role in stabilizing the ankle and maintaining balance during activities such as forward reaching. Dean et al. (1999) and Sivakumar et al. (2019) have highlighted the importance of this muscle in post-stroke rehabilitation. Dean et al. (1999) demonstrated that targeted exercises can enhance tibialis anterior activation, improving functional outcomes. Similarly, Sivakumar et al. (2019) found that modifying the sitting surface during reaching tasks can influence tibialis anterior muscle contractions, suggesting that environmental adjustments can optimize rehabilitation efforts. He discovered that reaching from sitting on an unstable surface provoked greater tibialis anterior contraction in the paretic lower extremity [10,11].

The present study is one among the few studies that have explored the effect of reaching in sitting on the quadriceps, specifically rectus femoris muscle activation. Nakamura et al. (2021) stated that notably rectus femoris activation started significantly earlier under the movable seating condition suggesting that the movable seat surface requires stronger knee stability during forward-reaching tasks [14]. Kim et al. (2019) reported that sitting and reaching have had major effects on two-joint muscles like the rectus femoris than single-joint muscles like the tibialis anterior. The activity was modified by the direction of reaching and position of the hip joint [5].

The manner of weight transfers and shifts during forward-reaching tasks differed between the experimental and control groups. In the experimental group, participants performed the reaching task with only the paretic foot grounded, forcing the paretic limb to stabilize the body. In contrast, the control group performed the same task with both feet supported. Previous studies have shown that the distribution of weight and foot support significantly impacts muscle activation in stroke rehabilitation. By grounding only, the paretic foot, the experimental setup aimed to challenge the paretic limb more, potentially leading to greater muscle activation and functional improvement [8,9].

Limitations

The forward-reaching distance of 1.5 times arm length post-stroke required some assistance to attain, which might have affected the study's outcomes. The study focused specifically on strokes occurring in the MCA territory, potentially restricting the applicability of the results to different stroke types or vascular syndromes like the posterior cerebral artery and anterior cerebral artery.

## Conclusions

Postural control strategies could be effectively used to provoke muscle contraction in patients with hemiplegia. Such tasks can be used to induce contractions of the lower extremities muscles because the lower extremities help control the forward weight shifts during reaching tasks while seated. Forcing the use of paretic lower extremity could be a better strategy to improve motor control. In this study, we removed the unaffected lower extremity from support, forcing the use of the paretic lower extremity to control the forward momentum while reaching forward. This strategy showed an increase in muscle contraction in the paretic lower extremity. It is not necessary for patients to pay attention to their lower extremities in order to trigger contractions because lower extremity contractions are reflexively elicited as part of the postural control set. Patients with reduced motor control may benefit from such activities, particularly those who are in the early subacute phase of a stroke. Consequently, we propose that a therapeutic strategy to induce lower extremity muscle contraction in patients with weak motor control and in the early sub-acute phase of stroke could involve sitting and reaching with only the paretic lower extremity grounded.
